# Whale-optimized LSTM networks for enhanced automatic text summarization

**DOI:** 10.3389/frai.2024.1399168

**Published:** 2024-08-29

**Authors:** Bharathi Mohan Gurusamy, Prasanna Kumar Rangarajan, Ali Altalbe

**Affiliations:** ^1^Department of Computer Science and Engineering, Amrita School of Computing, Amrita Vishwa Vidyapeetham, Chennai, India; ^2^Department of Computer Engineering, Prince Sattam bin Abdulaziz University, Alkharj, Saudi Arabia; ^3^Faculty of Computing and Information Technology, King Abdulaziz University, Jeddah, Saudi Arabia

**Keywords:** LSTM, Whale Optimization Algorithm, summarization, optimization, Auto Encoded

## Abstract

Automatic text summarization is a cornerstone of natural language processing, yet existing methods often struggle to maintain contextual integrity and capture nuanced sentence relationships. Introducing the Optimized Auto Encoded Long Short-Term Memory Network (OAELSTM), enhanced by the Whale Optimization Algorithm (WOA), offers a novel approach to this challenge. Existing summarization models frequently produce summaries that are either too generic or disjointed, failing to preserve the essential content. The OAELSTM model, integrating deep LSTM layers and autoencoder mechanisms, focuses on extracting key phrases and concepts, ensuring that summaries are both informative and coherent. WOA fine-tunes the model’s parameters, enhancing its precision and efficiency. Evaluation on datasets like CNN/Daily Mail and Gigaword demonstrates the model’s superiority over existing approaches. It achieves a ROUGE Score of 0.456, an accuracy rate of 84.47%, and a specificity score of 0.3244, all within an efficient processing time of 4,341.95 s.

## 1 Introduction

Over the past decade, text summarization has predominantly been a manual process, often time-consuming and subject to individual biases. The surge in the volume of information available online and from various sources has rendered manual summarization increasingly impractical. In the current era of information overload, automatic summarization has become essential for efficiently managing and distilling extensive text data.

Document summarization involves condensing text while retaining its crucial content, making it vital for effective information consumption. Whether extracting a summary from a single document or synthesizing multiple documents, summarization highlights key content, simplifying overall comprehension. Text summarization encompasses acquiring textual documents, processing the content, and delivering necessary information in a concise, user-friendly format. This essential process in modern information handling is broadly categorized into two types: abstractive and extractive summarization (Yang et al., 2019).

Abstractive summarization leverages Natural Language Processing (NLP) techniques to parse, reduce words, and generate summaries that encapsulate the original text’s core ideas in a new form. Conversely, extractive summarization, known for its flexibility and time efficiency, involves analyzing sentences in matrix forms and identifying key sentences through feature vectors—n-dimensional numerical representations of text objects (Mohan and Kumar, 2023). The objective of extractive summarization is to select sentences that align with specific requirements, effectively reducing text content while preserving its main message. Single document summarization focuses on condensing individual texts, whereas multi document summarization aims to synthesize and shorten a collection of similar documents into a cohesive summary. Despite their straightforward objectives, these tasks are complex and challenging to execute to the desired standards.

The advent of deep learning techniques has driven remarkable advancements in NLP. Text summarization, along with other tasks such as text translation and sentiment analysis, has significantly benefited from deep neural network models. These contemporary summarization approaches often utilize a sequence-to-sequence framework, typically an encoder–decoder model comprising neural networks trained on both input and output. Deep neural networks, leveraging large datasets, have demonstrated substantial improvements in summarization results.

Addressing these challenges and opportunities, this research introduces a groundbreaking system: the Optimized Auto Encoded Long Short-Term Memory Network (OAELSTM), enhanced by the Whale Optimization Algorithm (WOA). This innovative approach combines the robust feature extraction capabilities of Long Short-Term Memory (LSTM) networks with the data compression and efficiency benefits of autoencoders. The OAELSTM model is particularly adept at processing and synthesizing complex textual data into concise summaries.

The inclusion of WOA introduces a significant advancement in the approach. WOA is preferred over other benchmark metaheuristic algorithms like particle swarm optimization, gray fox optimization for the following reasons. Inspired by the bubble-net feeding behavior of humpback whales, WOA offers a unique biological basis that mimics natural behaviors not found in other algorithms, potentially providing advantages in handling specific optimization challenges. Moreover, WOA inherently balances exploration and exploitation, crucial for optimizing complex models like neural networks, which allows it to efficiently discover optimal solutions. Its randomized approach to solution updates, akin to the diving behavior of whales, may also lead to better convergence rates and solution quality compared to other swarm-based optimization algorithms. These factors collectively make WOA a robust choice for enhancing the efficiency and effectiveness of text summarization models.

Departing from traditional sentence-level summarization, the OAELSTM model focuses on extracting and rearticulating key phrases and pivotal concepts. This approach enhances the relevance and informativeness of generated summaries while effectively addressing common issues such as repetition and redundancy often encountered in standard summarization techniques. By prioritizing meaningful content extraction and employing advanced neural network optimization, this system sets a new benchmark in automatic text summarization.

The Important contribution of this paper as follows:

- Introduction of the Optimized Auto Encoded Long short-term memory for text summarization, blending the robust feature extraction of LSTMs with the efficiency of autoencoders, marking a significant advancement in the field of automatic text summarization.
- Innovative application of the Whale Optimization Algorithm to optimize the OAELSTM model, a pioneering approach in natural language processing that enhances accuracy and efficiency in processing complex text.
- Shift from traditional sentence-level summarization to a focus on extracting and synthesizing key phrases and concepts, leading to more concise and content-rich summaries.
- Demonstrated superior performance of the OAELSTM model through rigorous evaluation on challenging datasets like Daily Mail and Gigaword, outperforming existing models as evidenced by ROUGE metric benchmarks.

The organization of this paper is structured as follows: Section 2 discusses related works, highlighting existing research and approaches in text summarization. Section 3 details the methodology employed, including the implementation of the WOA for feature selection and model optimization for summarization. Section 4 presents the results and discussions, where the effectiveness of the proposed approach is analyzed and compared with existing methods. Section 5 concludes the paper with a summary of findings and outlines avenues for future research and enhancements in text summarization techniques.

## 2 Related works

The field of text summarization has been predominantly driven by extractive techniques, where the focus has been on identifying and reproducing key sentences and phrases from source documents. This backdrop provides a context for exploration into the OAELSTM with WOA.

Ramachandran et al. (2022) introduced an autonomous approach for document summarization that emphasizes discourse coherence and keyword extraction through clustering algorithms. Their results demonstrate the efficiency of their method in creating clustered summaries. However, their approach may encounter scalability issues when applied to large-scale document collections, and the subjective nature of discourse coherence assessment. Complementing this, Yadav et al. (2022) offer a comprehensive overview of various text summarizing approaches and techniques, providing a broad perspective on the status and future directions of text summarization. In a similar vein, Latha et al. (2022) utilized NLP techniques, particularly Hugging Face transformers, to summarize video transcripts. This approach underlines the efficiency of NLP in extracting key patterns for summarization. On the abstractive front, Dugar et al. (2022) tackled unsupervised text summarization using an adversarial autoencoder model, combining K-Means clustering with language models for summary generation, model limits adaptability to less structured or specialized domains.

Pawar et al. (2022) explored an extractive text summarization technique using sentence clustering, employing Jaccard and Cosine similarity methods and the model depends on the similarity measure alone. While their approach shares similarities with the presented work in terms of clustering, the summarization technique differs. Ranganathan and Abuka (2022) leveraged the Text-to-Text Transfer Transformer (T5) model for summarization, focusing on the University of California, Irvine’s drug reviews dataset, but the potential bias and capturing drug specific details are limitations. Pan et al. (2022) proposed a context-aware BERT ranking framework using abstractive summarization to enhance text semantics, while Jain and Saini (2023) evaluated the effects of Extractive and Abstractive Summarization on text document classification. They utilized Gensim and Pegasus summarizers for extractive and abstractive summaries, respectively, highlighting the nuanced effects of NLP preprocessing in deep learning.

Further, Latief et al. (2023) critically evaluated the impact of preprocessing techniques like tokenization and case folding on sentence interpretability in deep learning models. Kuyumcu et al. (2019) challenged the norm by assessing the performance of a fastText classifier on Turkish text without preprocessing, demonstrating robust classification capabilities even with raw text. Shakil and Alam (2022) have made notable strides in the classification of hate speech by combining Convolutional Neural Networks (CNN) with NLP. Their research delves into the nuances of content moderation, focusing on creating deep learning models that are not only effective but also interpretable and explainable. Arora and Kansal (2019) contribute to this field by focusing on sentiment analysis using deep learning techniques to process raw Twitter data. Their work highlights the importance of text normalization in handling unstructured data, a challenge that is also central to proposed research.

Nadimi-Shahraki et al. (2022) propose an enhanced version of the WOA, designed to tackle global optimization problems. The potential of this enhanced WOA in the realm of feature selection for text summarization is noteworthy. The adaptation of such advanced optimization techniques could significantly improve the efficiency and accuracy of summarization models. Kundu et al. (2022) presents a study on an Altruistic WOA, demonstrating its superior accuracy and feature selection capabilities. The insights from this study could be particularly insightful for text summarization tasks, where selecting the most relevant features from the text is crucial for generating concise and coherent summaries.

Riyahi et al. (2022) demonstrates a WOA that effectively reduces the number of features while maintaining or even enhancing classification accuracy. This approach is directly relevant to the presented work, as it aligns with the objective of optimizing feature selection in text summarization, ensuring that our model captures the essence of the text without unnecessary complexity. Sun et al. (2023) developed a two-stage feature selection model using a binary WOA. The methodology proposed in this study could be applied to the feature selection process in text summarization, potentially enhancing the capability of proposed model to distill and summarize large volumes of textual data efficiently. Too et al. (2021) propose a new variant of WOA for high-dimensional feature selection. This variant could be particularly beneficial in summarizing complex text data, where dealing with high-dimensional feature spaces is a common challenge.

Malarselvi and Pandian (2023) introduce a multi-layered CNN model designed for feature representation in text summarization. This model is adept at extracting both linear and non-linear information, demonstrating the growing sophistication in using CNNs for complex text analysis tasks. Yu et al. (2021) present LenAtten, a novel model that notably improves length controllability and ROGUE scores in summarization tasks. Tested on the CNN/Daily Mail dataset, their approach provides insights into enhancing the precision and effectiveness of summary generation. Zhang et al. (2019) propose a novel pretraining-based encoder–decoder framework, generating output sequences for summarization. Their model achieves state-of-the-art results on CNN/Daily Mail and New York Times datasets, highlighting the potential of advanced encoder–decoder structures in text summarization.

Song et al. (2018) report a framework that outperforms existing state-of-the-art models in terms of semantic and syntactic structure. The effectiveness of their model on datasets including CNN/Daily Mail underscores the importance of syntactic and semantic understanding in creating accurate and coherent summaries. Aliakbarpour et al. (2021) propose a novel abstractive summarization model that utilizes a combination of CNN and LSTM with an auxiliary attention mechanism. In their research, Gurusamy et al. (2023) have made significant strides in automatic text summarization by developing a hybrid model. This model integrates extractive methods, notably Semantic Latent Dirichlet Allocation (Semantic LDA) and Sentence Concept Mapping, with a transformer-based abstractive approach. Bharathi Mohan et al. (2023) delves into the comparison between BERT and GPT-2 in the context of text summarization, focusing on their performance in processing and summarizing multiple documents.

Existing text summarization methods face numerous challenges. Extractive techniques produce summaries lacking deeper context. Clustering-based approaches struggle with scalability and coherence. Graph-based methods are computationally heavy and struggle with informativeness versus redundancy. Adversarial models may not adapt to specialized domains and miss nuanced features. Similarity-focused techniques overlook semantic relationships. Abstractive models are resource-intensive and can introduce biases. Preprocessing techniques reduce interpretability with noisy data. Optimization methods need tuning for text complexity. CNN-based models and frameworks may not generalize well. Semantic methods are computationally demanding and prone to overfitting. WOA addresses these issues by efficiently selecting key features, reducing text dimensionality, and maintaining essential content. It is scalable, handles large tasks efficiently, adapts to various text types, and integrates well with other methods. WOA ensures coherent summaries by balancing informativeness and redundancy, making it a robust and adaptable solution for improving text summarization quality.

## 3 Methodology

### 3.1 Overall architecture

**Figure 1:**
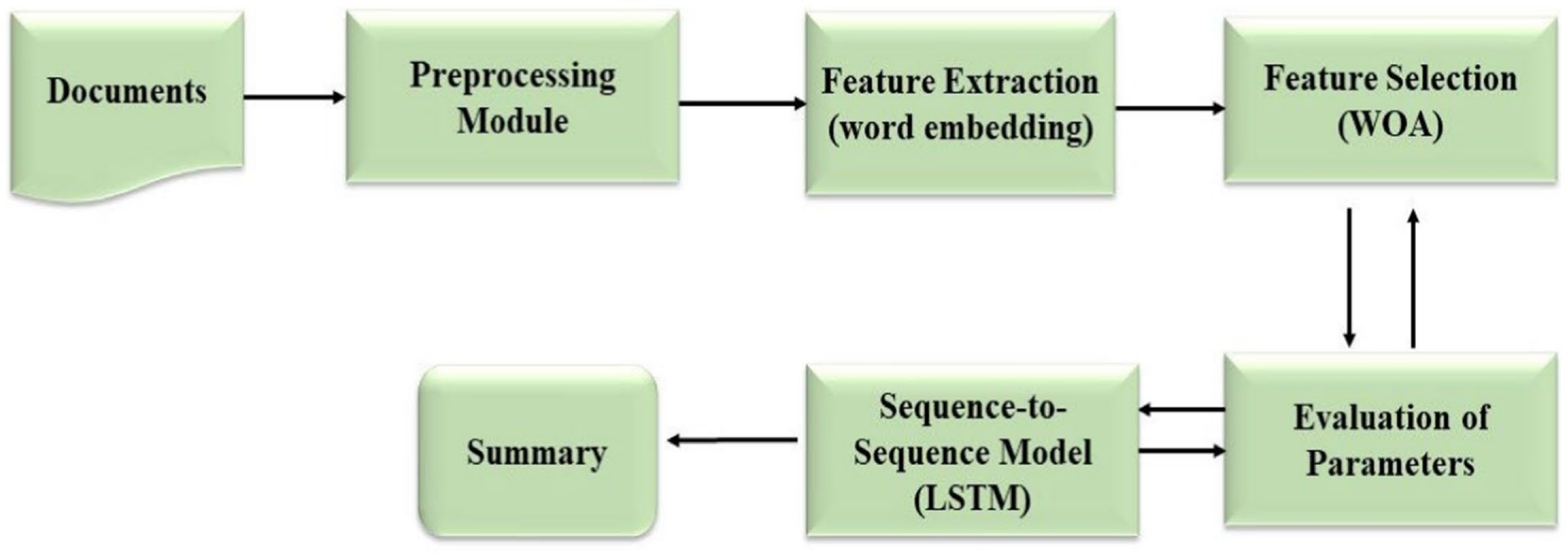
Overall architecture for proposed system.

Figure 1 illustrates the architecture of the proposed model of text summarization system OAELSTM, employs a sophisticated architecture that progresses from the initial input of raw text to the generation of concise summaries. The system first preprocesses the text, where it undergoes tokenization and normalization to structure the data for feature extraction. Subsequently, meaningful features are distilled from the text, encapsulating its linguistic nuances. These features are then refined through the Whale Optimization Algorithm (WOA), which selects the most salient attributes essential for summarization. The core of the system is the LSTM network, designed to understand and preserve the context within the text. Integral to this network is an iterative optimization loop that fine-tunes the model parameters, enhancing the quality of the summarization. The culmination of this process is the output summary, which is meticulously evaluated for its fidelity to the original text using the ROUGE scoring metric, accuracy, and specificity. This end-to-end architecture is crafted to ensure that the final summary is not only succinct but also retains the integral meaning of the source text.

### 3.2 Data collection and preprocessing

The study utilizes a comprehensive dataset, CNN/Daily Mail^1^ and Gigaword,^2^ comprising a collection of document-summary pairs. The CNN/Daily Mail dataset, comprising news articles and human-written summaries, includes over 287,000 samples. The Gigaword dataset, derived from various news sources, consists of around 3.8 million training sets, alongside substantial testing, and validation sets.

**Figure 2:**
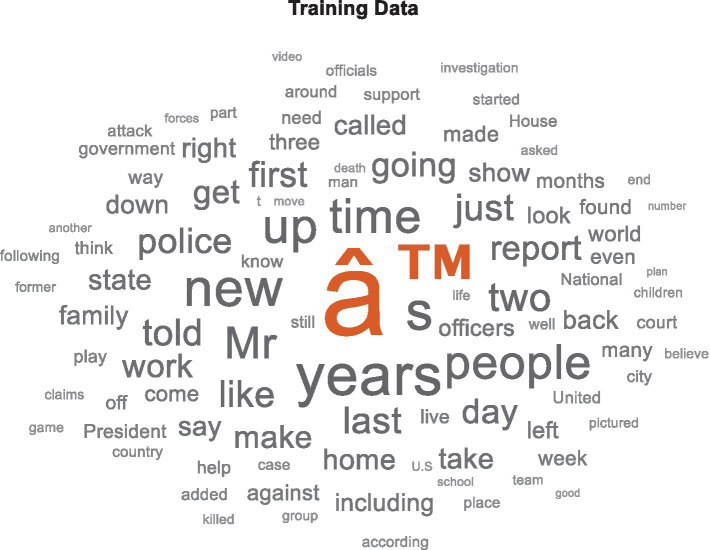
Word cloud for data processing.

The dataset serves as the foundational corpus for training and evaluating the proposed OAELSTM model. Initially, a table is created with two columns: Description and Category, represents the document content and its associated summary, respectively. The tabular format facilitates easier manipulation and processing of the data. To provide an overview of the training data, generate a word cloud visualization, which helps in identifying the most frequent terms within the dataset shown in Figure 2.

Effective preprocessing is paramount in preparing of the dataset for the OAELSTM model. These steps ensure that the input data is uniform and optimized for subsequent analysis:

- Tokenization: Each document is broken down into smaller units, or tokens, typically words or phrases, for systematic analysis.
- Case Normalization: All tokens are converted to lowercase to ensure consistency and avoid treating the same words with different capitalizations as distinct.
- Punctuation Removal: Punctuation marks are removed to streamline the text and focus on the core content.
- Stemming: Tokens are reduced to their root forms, allowing the model to process and recognize the fundamental meanings of words.

### 3.3 Feature representation through word embedding

In the methodology, MATLAB’s trainWordEmbedding function is utilized for word embedding training with the Continuous Bag of Words (CBOW) approach. Parameters include a 30-dimensional embedding dimension to balance semantic richness and computational efficiency, training conducted over 50 epochs to capture intricate semantic patterns, a context window size of 5 to consider word associations, an initial learning rate set at 0.05 for effective parameter updates, and a minimum word frequency count of 5 to filter out rare terms and noise from the vocabulary.

#### 3.3.1 Word embedding training

The training of the word embedding is a crucial aspect of the proposed methodology. We set the embedding dimension to 30, aiming to balance the semantic richness of the word representations with computational efficiency. The embedding dimension directly impacts the semantic representation of words. A higher dimensionality can capture more nuanced relationships between words but requires more computational resources. Conversely, a lower dimensionality sacrifices some semantic granularity but improves computational efficiency. The training is conducted over 50 epochs to ensure comprehensive learning of the relationships between words in the dataset. More epochs generally allow for deeper learning and refinement of word embeddings. The choice of 50 epochs ensures comprehensive learning of word relationships without overfitting to the training data.

Utilizing MATLAB’s trainWordEmbedding function, the approach can effectively train the word embeddings. This function is specifically designed for creating word embeddings, providing us with the tools to capture the nuanced semantic relationships inherent in the text. During this process, we maintain streamlined execution by disabling verbosity, which not only enhances the efficiency of the training but also minimizes distractions, a key consideration when dealing with large datasets.

#### 3.3.2 Preparing document sequences

Post-training, the focus shifts to standardizing the length of each document to 40 tokens. This uniformity in sequence length, achieved using a custom document function, is crucial for ensuring consistent input lengths for neural network processing. Following the truncation, these documents are converted into sequences of word vectors using the trained embedding (emb). This conversion is essential to translate the text into a numerical format, rendering it suitable for analysis by neural network models.

#### 3.3.3 Document sequence processing

A significant aspect of the methodology involves processing each document to transform it into a sequence of word vectors. This process is essential to align the textual data with the trained word embedding, ensuring that every word in a document is represented in its vector form.

a. Word Selection

The process begins by filtering words in a document, retaining only those found in the trained word embedding. This ensures the inclusion of words that carry meaningful semantic information.

b. Conversion to Vectors

Selected words are then transformed into their vector representations, converting textual data into a numerical format suitable for neural network analysis.

c. Optimization for Efficiency

To handle large datasets efficiently, the process is optimized for parallel processing, allowing multiple documents to be prepared simultaneously, thus enhancing speed and efficiency.

d. Resulting Data Format

The outcome is a sequence for each document where words are represented by their vector embeddings, ensuring consistent data representation for deep learning models.

e. Data Padding

Finally, sequences are padded to a uniform length, essential for batch processing in neural networks, ensuring stable and reliable model performance.

#### 3.3.4 Dataset partitioning and parameter setting

##### 3.3.4.1 Data preparation for model training and validation

In this phase of the research process, focus shifts to preparing the dataset for both training and validation. This step is crucial for evaluating the performance of the model and ensuring its generalizability.

a. Loading the Training Data

The process begins by loading the training data (XTrain), which has been previously processed and prepared for the model.

b. Partitioning the Dataset

To assess the model’s performance accurately, we allocate a portion of the data for validation. Using the hold-out method, we set aside 20% of the data as a validation set. This partitioning is achieved using MATLAB’s cvpartition function with a hold-out ratio (ho) of 0.2, ensuring that the model is tested on unseen data, a critical factor for evaluating its robustness and predictive power.

##### 3.3.4.2 Parameter setting for the model

The process of setting parameters is essential for the configuration and optimization of the proposed model.

a. Parameter Initialization

We initialize several key parameters, including the number of search agents (N) set to 10 and the maximum number of iterations (max_Iter) set to 100. These parameters are integral to the operation of the model, impacting its learning and optimization capabilities.

b. Summary Extraction for Demonstration

To showcase the functionality of the model, we extract a summary from the first category in the dataset. We use the function for summary extraction, specifying the size of the summary as 6 units and ordering it by position. This extracted summary, obtained from the model’s vocabulary, serves to demonstrate the summarization capability of the system.

c. Preparation for Optimization

The summary extracted is then used in conjunction with the training data (XTrain) for the subsequent steps in the model’s training and optimization process.

### 3.4 Feature selection using WOA

In the study, an algorithm denoted as WOA_Opt_Feature ) was implemented to optimize feature selection using the Whale Optimization Algorithm (WOA) (Algorithm 1). This algorithm is integral to identifying the most effective features for the text summarization model. The algorithm is designed to perform feature optimization through the principles of WOA. It operates by simulating the social and hunting behavior of humpback whales, a process that involves iteratively updating the positions of search agents (potential solutions) within the search space.

#### ALGORITHM 1: WOA_Opt_Feature


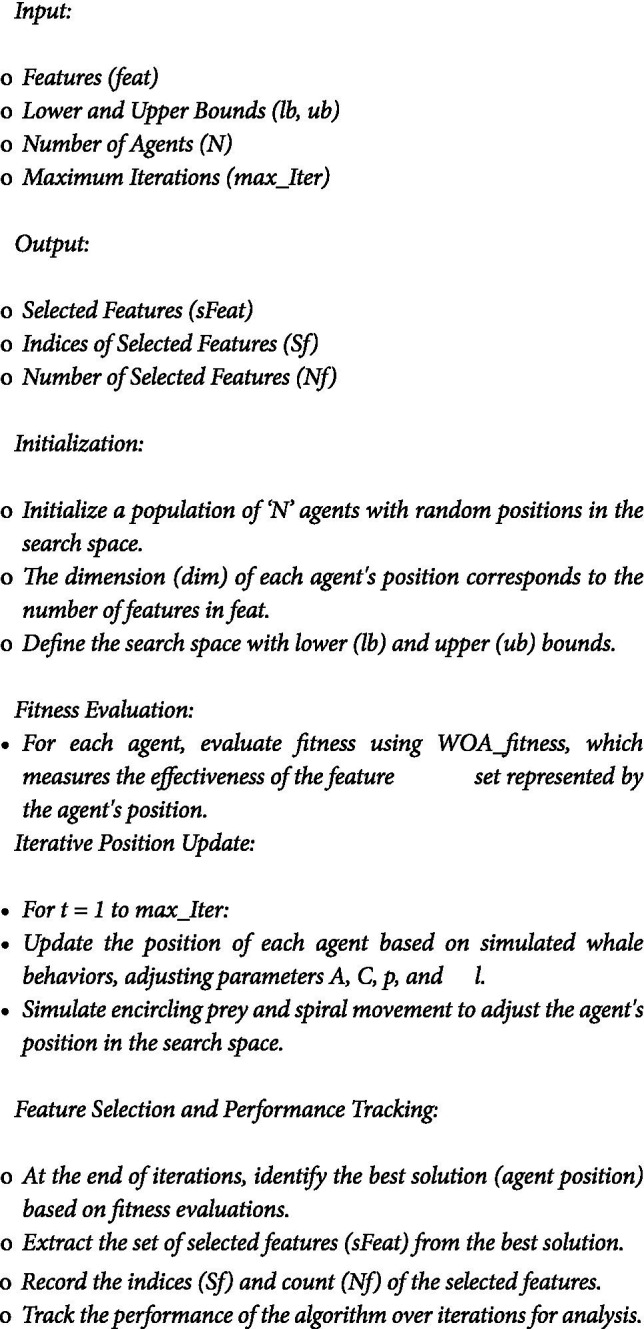


### 3.5 Evaluation of feature set effectiveness

In the study, a two-part algorithm is employed to evaluate the effectiveness of feature sets selected by the Whale Optimization Algorithm for the text summarization model. This evaluation is crucial in identifying the most relevant and impactful features for summarization tasks. The effectiveness of o feature selection discussed in Section 4.2.

- Fitness Function: Evaluating Feature Sets

The first part of the algorithm is the fitness function, WOA_fitness (Algorithm 1.1). This function plays a pivotal role in the Whale Optimization Algorithm (WOA) by assessing the quality of each feature set.

#### ALGORITHM 1.1: Fitness Evaluation in WOA_Opt_Feature


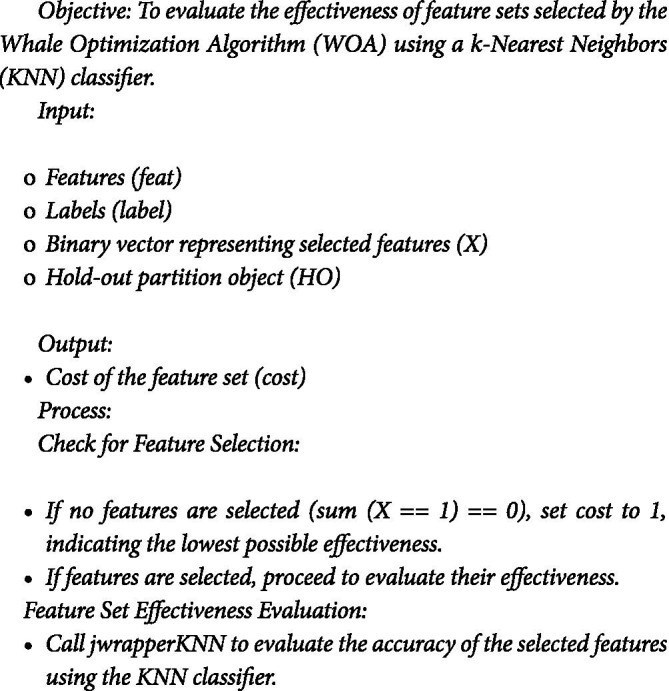


*Calculate the cost as the inverse of the accuracy obtained from jwrapperKNN.*

b. Evaluate the fitness function

The second part of the algorithm is evaluating the fitness function using KNN classifier. To evaluate the effectiveness of a feature set, the fitness function utilizes a wrapper function for the k-Nearest Neighbors (KNN) classifier, named jwrapperKNN and Algorithm 1.2 outlines the fitness function. The process involves using selected features (sFeat) and corresponding labels (label) with a hold-out partition object (HO) to evaluate a KNN classifier. Parameters are set with *k* = 5 for the number of nearest neighbors. Data is split into training (xtrain, ytrain) and validation (xvalid, yvalid) sets using HO. The KNN model is trained on xtrain and ytrain, then used to predict labels for xvalid. Accuracy is calculated as the proportion of correct predictions, from which the error rate (error = 1 − Acc) is derived to assess classifier performance.

#### ALGORITHM 1.2: Wrapper Function for KNN Classifier: jwrapperKNN


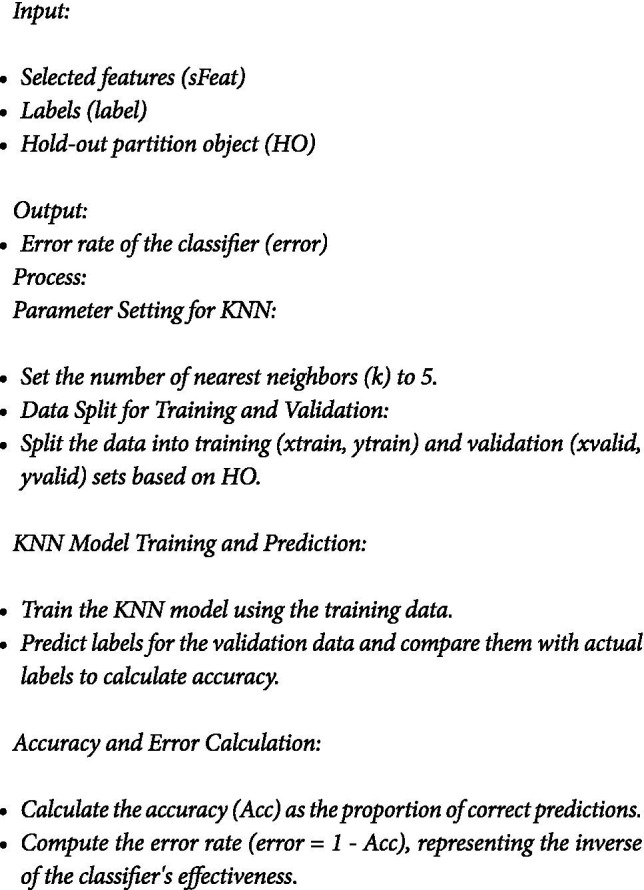


#### 3.5.1 Parameter and objective space in WOA optimization

**Figure 3:**
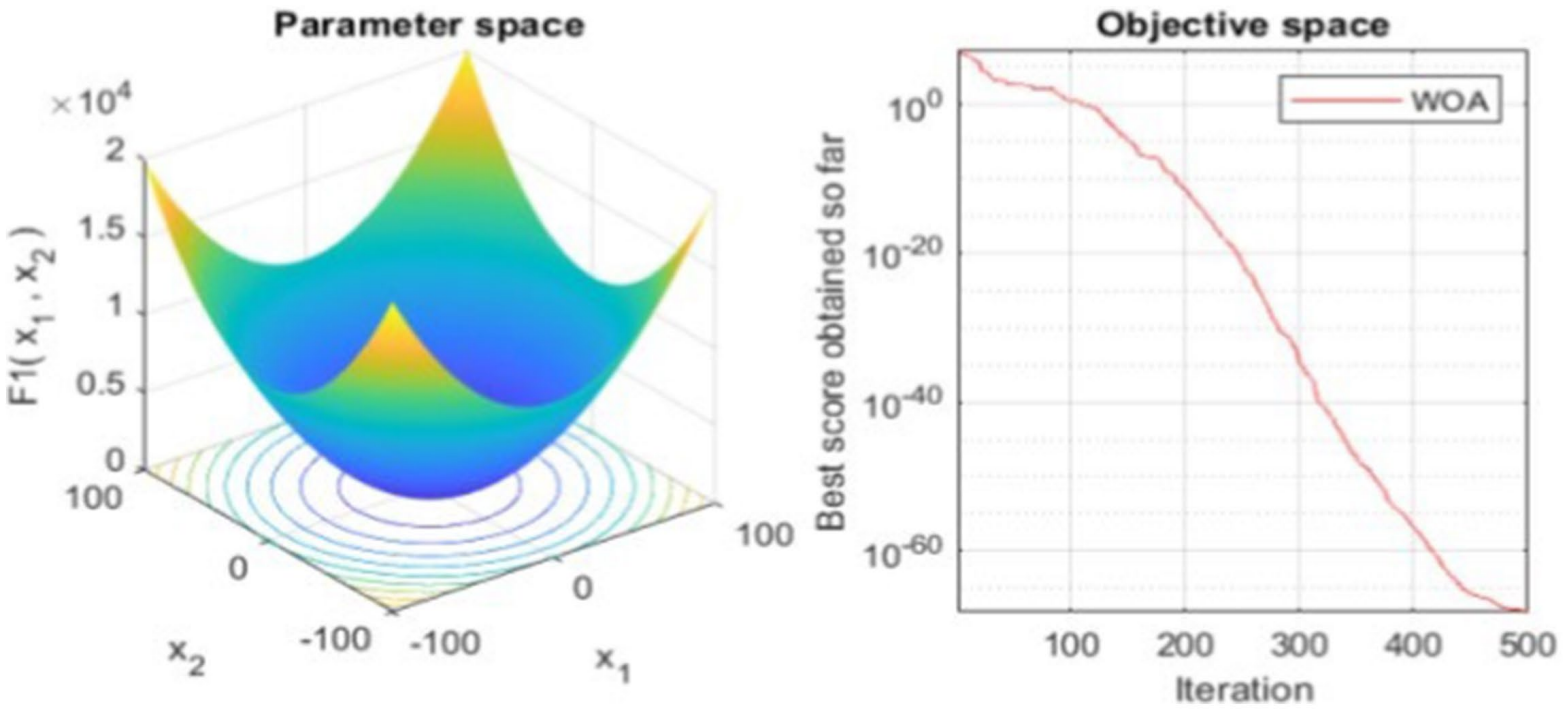
Parameter space vs. objective space in WOA optimization.

To illustrate the optimization process, we plot the parameter space and the objective space. The parameter space plot visualizes the function landscape that the WOA navigates, providing insights into the complexity of the optimization task. The objective space plot, drawn in a semilogarithmic scale, shows the progression of the algorithm over iterations, highlighting the improvement in the solution with each step The outputs of this process, including the best solution and the optimal value of the objective function, are displayed, providing clear evidence of the effectiveness of WOA in optimizing the model. The performance of the algorithm over iterations is tracked using a convergence curve shown in Figure 3 and detailed analysis done in Section 4.1.

### 3.6 Summarization using sequence to sequence model LSTM

#### 3.6.1 Data preparation for training and testing

Optimized Training Data (XTrain1): This dataset, which has undergone feature selection via WOA, provides a refined set of features for the model, ensuring that only the most relevant information is used for training and testing. This enhances the model’s ability to focus on significant textual patterns.

Training and Test Set Partitioning: Splitting the data into training (XTrain) and test (XTest) sets ensures that the model is evaluated on unseen data, a key aspect for assessing its generalizability.

#### 3.6.2 Model architecture for text summarization

In developing the text summarization model, a carefully designed architecture leverages the strengths of neural networks in processing and interpreting natural language data.

a. Sequence Input Layer

The foundation of the model is the sequence input layer, specifically tailored to handle sequences of data. In this application, this involves processing sequences of word vectors derived from trained word embeddings. This layer effectively captures the sequential nature of text data, setting the stage for deeper analysis.

b. Intermediate Layers

Following the input layer, the model comprises a series of intermediate layers, each serving a distinct purpose:

RELU Activation Layers: These layers introduce non-linearity into the model, enabling it to learn complex patterns in the data.

Flattening Layer: This layer flattens the output from the previous layers into a format suitable for input into the LSTM layers, ensuring seamless data flow.

LSTM Layers with Dropout: We incorporate Long Short-Term Memory (LSTM) layers, each with 200 hidden units. These layers are pivotal in capturing long-range dependencies within the text, a critical aspect of understanding and summarizing content. The inclusion of dropout layers helps in preventing overfitting, a common challenge in deep learning models.

c. Output Layers

The culmination of the model is in its output layers, which consist of:

Softmax Layer: This layer computes the probability distribution across 6,973 classes, corresponding to the potential summaries. It transforms the output of the LSTM layers into a probabilistic format, indicating the likelihood of each class being the correct summary.

Classification Layer: Serving as the final layer, it is responsible for the actual classification task, determining the most probable summary based on the probabilities provided by the softmax layer.

#### 3.6.3 Training setup for the text summarization model

In developing the text summarization model, the training setup has been meticulously configured to optimize the learning process and ensure the model effectively captures the nuances of the data.

a. Adam Optimizer

The ‘adam’ optimizer used for training the model. Adam, short for Adaptive Moment Estimation, is a popular optimization algorithm in deep learning because it combines the advantages of two other extensions of stochastic gradient descent: Adaptive Gradient Algorithm (AdaGrad) and Root Mean Square Propagation (RMSProp). Adam is known for its effectiveness in handling sparse gradients and its adaptive learning rate capabilities, making it particularly suited for complex tasks like text summarization.

b. Maximum Epochs

The training process is set to run for a substantial number of epochs, specifically 1,500. This high epoch count is chosen to ensure that the model undergoes comprehensive learning, thoroughly adjusting and refining its weights in response to the intricacies of the training data. Training is crucial for deep learning models, as it allows for the gradual and detailed extraction of features and patterns from the text.

c. Initial Learning Rate from WOA

A novel aspect of the training setup is the use of the best score obtained from the Whale Optimization Algorithm (WOA) as the initial learning rate. This approach integrates the insights from the feature selection process directly into the model’s training. By adapting the learning rate based on the performance of feature sets optimized by WOA, we align the model’s learning process more closely with the characteristics of the most effective features. This synergy enhances the overall efficacy of the model.

d. Training Data Subset

The model is trained on a carefully selected subset of the processed dataset (XTrain). This subset comprises sequences of text data, pre-processed and structured for efficient learning. The corresponding labels (Feature selected or not), which categorize the data, are used to guide the supervised learning process. These labels play a critical role in shaping the model’s understanding and generation of summaries. Each piece of data within this subset is associated with specific labels that guide the supervised learning process. These labels serve crucial roles in shaping how the model understands and generates summaries. The process of creating this subset involves filtering XTrain based on these labels to construct a balanced and representative training set.

#### 3.6.4 Model evaluation

After the completion of the training phase, we conducted a comprehensive evaluation of the text summarization model. This evaluation aimed to assess the model’s performance in accurately summarizing text, both quantitatively and qualitatively.

a. Classification Process

The proposed system employed the trained model (to perform classification tasks on both the training dataset (XTrain) and the test dataset (XTest). This step is crucial to understand how well the model performs on data it has seen (training data) and on new, unseen data (test data).

b. Accuracy and Recall Assessment

The model’s predictions were systematically compared against the original labels of the datasets. This comparison allowed us to evaluate two key metrics: accuracy, which measures the proportion of correctly predicted summaries, and recall, which assesses how many relevant summaries were correctly identified by the model.

## 4 Results and discussion

### 4.1 Parameter space and object space analysis

In the results, Figure 1 play a crucial role in visualizing the efficacy of the proposed WOA based algorithm WOA_Opt_Feature applied to the text summarization model.

Figure 3 illustrates the complex optimization landscape that the WOA navigates. The three-dimensional surface plot, with axes representing the parameters being optimized, depicts the cost function landscape. The multi-modal nature of this landscape, with its numerous local minima, is evident and underscores the robustness of the WOA in seeking out the global minimum. The valleys and contours on this plot provide a visual representation of the algorithm’s search strategy, reflecting the intricate exploration and exploitation balance maintained throughout the optimization process.

Figure 3 also presents the objective space through a semilogarithmic plot, capturing the proposed WOA’s iterative performance. The convergence curve showcases the algorithm’s efficiency in progressively refining the solution. Notably, the sharp decline in the curve during the initial iterations indicates rapid improvements in solution quality, which is a testament to the WOA’s capability for quick convergence. The flattening of the curve in later iterations suggests that the algorithm is approaching the optimal solution, with incremental gains as it fine-tunes the parameters.

The convergence curve, as detailed in Figure 3, is indicative of the optimization depth achieved by the WOA. The best score obtained and the corresponding position in the parameter space are significant outcomes that demonstrate the potential of the WOA in enhancing the model’s summarization performance. The plots collectively offer a comprehensive overview of the optimization journey, from the initial parameter setting to the final selection of an optimal feature set.

### 4.2 Feature selection impact on training data

In the process of refining the model, we applied the Whale Optimization Algorithm (WOA) to select the most relevant features from the training data. This section compares the training data before (XTrain) and after (XTrain1) feature selection to demonstrate the algorithm’s effectiveness.

a. Before Feature Selection: XTrain

**Figure 4:**
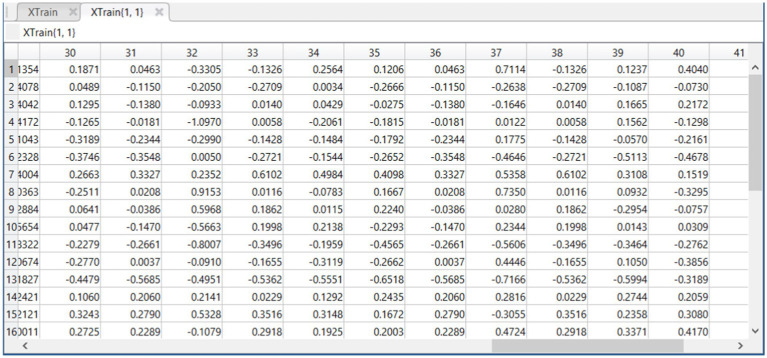
Feature matrix XTrain derived from dataset before feature selection.

The initial training data, visualized in Figure 4, contains a full set of features, represented by a multidimensional array where each column corresponds to a specific feature of the data. The diversity in the values reflects the comprehensive nature of the raw data set, which includes a wide range of information encapsulated in the various dimensions.

b. After Feature Selection: XTrain1

**Figure 5:**
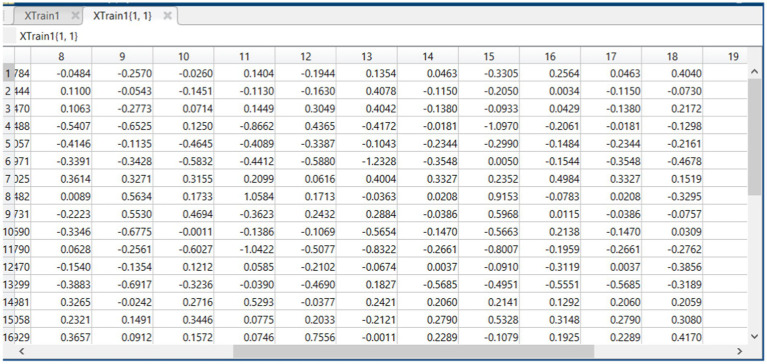
Feature matrix XTrain1 derived from dataset after feature selection.

Figure 5, which represents the training data after feature selection, shows a refined set of features. The reduction in dimensions is indicative of the WOA’s ability to identify and retain the most informative features, discarding those that contribute less to the summarization task’s performance. The resulting dataset is expected to be less noisy and more focused on the key aspects that contribute to generating accurate text summaries.

c. Interpretation of Results

By comparing the two figures, we can observe the dimensionality reduction achieved through the optimization process. The selective feature set in XTrain1 is anticipated to enhance the model’s learning efficiency by reducing the complexity of the input space and focusing on the quality rather than the quantity of the information provided during training.

The distilled feature set not only streamlines the training process but also is likely to improve the model’s generalizability by mitigating the risk of overfitting. With fewer, more impactful features, the model can better capture the essence of the text, resulting in more precise and coherent summaries. The results suggest that the proposed WOA has successfully optimized the feature space, potentially leading to improved summarization accuracy and a more robust model.

### 4.3 Quantitative analysis of proposed framework

#### 4.3.1 Rouge scores

The Rouge-N score evaluates the similarity between n-grams in the generated summary and a reference summary, with Rouge-1 specifically focusing on the overlap of individual words. This metric, crucial for assessing summarization quality, quantifies the extent of shared content, providing a clear measure of a summary’s accuracy and relevance. The Rouge-N score measures the overlap between the n-grams in the generated summary and the reference summary. For Rouge-1 (which measures the overlap of 1-gram or each word), the formula is shown in Equation 1:


(1) $$\mathrm{Rouge}:N=\frac{\begin{array}{l} \sum s\in \left\{\mathrm{Reference}\;\mathrm{Summaries}\right\}\\ \sum \mathrm{gramn}\in s\;\mathrm{Count}\left(\mathrm{gramn}\right) \end{array}}{\begin{array}{l} \sum s\in \left\{\mathrm{Reference}\;\mathrm{Summaries}\right\}\\ \sum \mathrm{gramn}\in s\;\mathrm{Countmatch}\left(\mathrm{gramn}\right)\; \end{array}}$$

Where Count_match_ (gram_n_) is the maximum number of n-grams co-occurring in a candidate summary and a set of reference summaries. Count (gram_n_) is the count of n-grams in the reference summaries.

**Figure 6:**
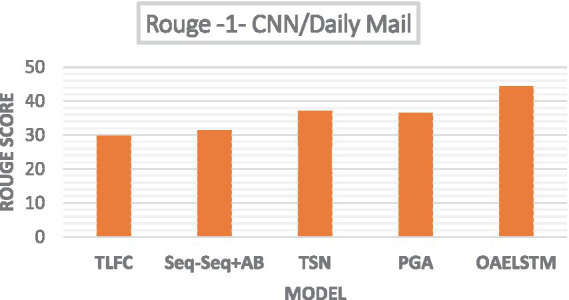
Rouge scores—CNN/Daily Mail dataset.

Figure 6 presents a bar chart comparing the Rouge scores of several text summarization algorithms such as Token Level Fact Correction (TLFC), Two Stage Network (TSN), Pointer Generator Algorithm (PGA) and Seq-Seq + Attention Baseline (S-S + AB) highlighting the performance of the proposed system. Our study’s comparative analysis on the CNN/Daily Mail dataset, as illustrated, reveals the OAELSTM system’s superior performance in automated text summarization. With a ROUGE-1 score of 44.34, the OAELSTM system outperforms other notable algorithms—TLFC (29.86), Seq-Seq + AB (31.48), TSN (37.16), and PGA (36.44)—underscoring its advanced capability in producing summaries that closely align with human reference standards.

**Figure 7:**
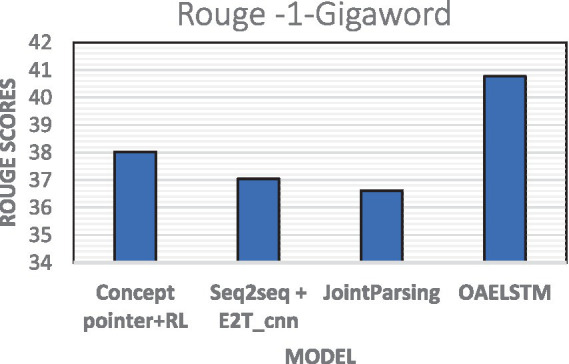
Rouge scores—Gigaword dataset.

The evaluation conducted on the Gigaword dataset demonstrated the effectiveness of proposed OAELSTM model, which achieved a ROUGE-1 score of 40.78 shown in Figure 7. This outperforms other summarization approaches, including Concept Pointer + RL (38.02), Seq2seq + E2T_cnn (37.04), and JointParsing (36.61). The higher score of OAELSTM indicates its superior capability in generating summaries that more closely mirror the reference texts.

#### 4.3.2 Specificity

Especially within the context of the approach using the OAELSTM model, Specificity is important because it measures the model’s ability to correctly identify and reject non-essential or irrelevant information when creating a summary. In the case of the OAELSTM model, which likely includes an optimization component (WOA) to refine feature selection, specificity helps to gauge how well the model has been tuned to prioritize the most significant features for summarization. A high specificity score would suggest that the model, after optimization, is adept at pinpointing and leveraging the most informative features while disregarding the less important ones.

Specificity: Also known as the True Negative Rate, it measures the proportion of actual negatives that are correctly identified given in Equation 2


(2) $$\mathrm{Specificity}=\frac{\mathrm{True}\;\mathrm{Negatives}}{\mathrm{True}\;\mathrm{Negatives}+\mathrm{False}\;\mathrm{Positives}}$$

Where True Negatives are the correctly identified negative cases and False Positives are the cases that were incorrectly predicted as positive but are negative.

**Figure 8:**
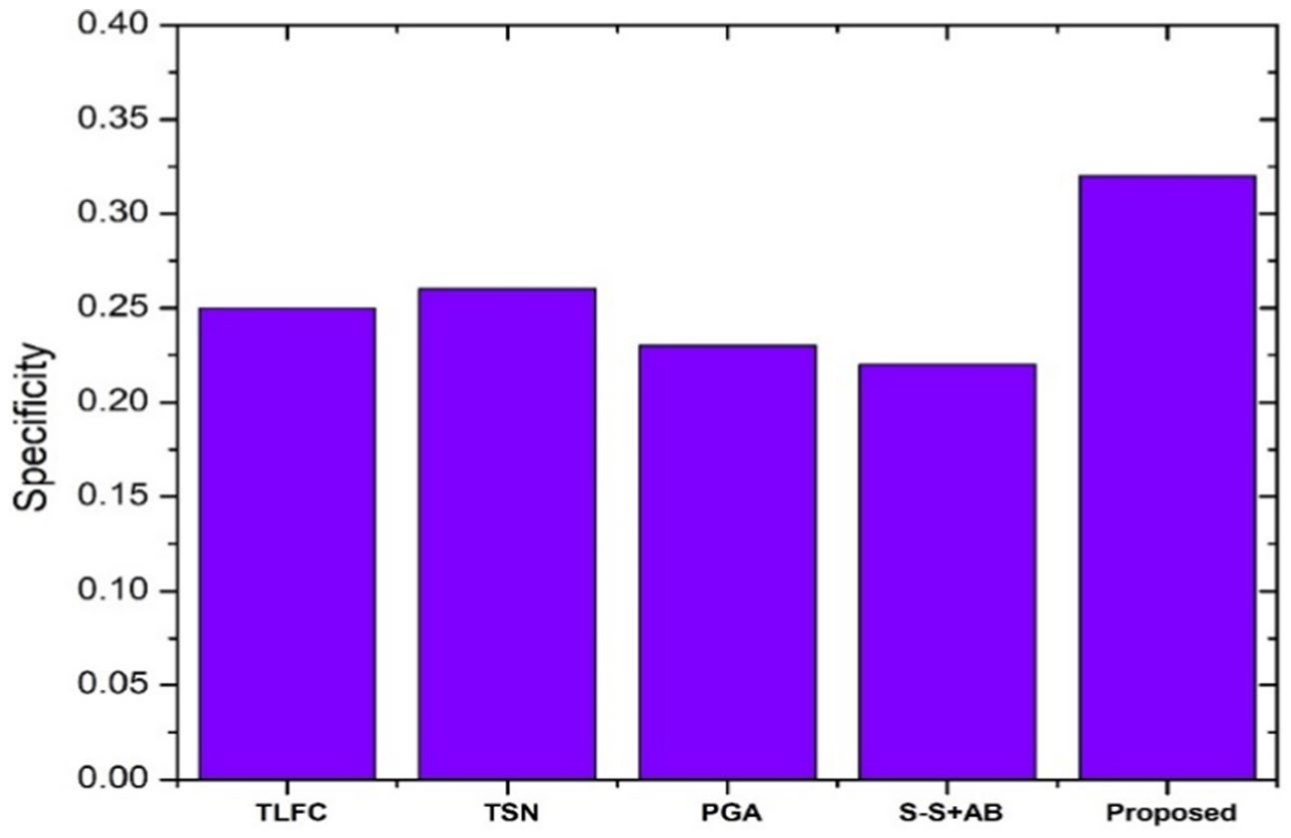
Specificity scores comparison.

Figure 8 exhibits the specificity scores of different summarization algorithms, emphasizing the performance of the proposed model on CNN/Daily Mail Dataset. The proposed system exhibits a notably higher specificity score, outshining its counterparts. This metric is indicative of the model’s ability to correctly include pertinent details in the summaries while excluding irrelevant information. Compared to other models like the Token-level fact correction and the Pointer generator, the proposed system’s advanced feature selection process likely contributes to its enhanced specificity, enabling it to focus more accurately on the essential content of the text.

#### 4.3.3 Accuracy

Accuracy measurement in text summarization is essential for evaluating how closely the model’s generated summaries match the expected outputs. It serves as a key metric for assessing the effectiveness of the summarization model, like OAELSTM, and is crucial for fine-tuning the model’s parameters and for benchmarking its performance against other models. High accuracy ensures that the summaries are reliable and reflective of the original text, thereby enhancing user trust and experience.

Accuracy is the ratio of correctly predicted observations to the total observations and is used for classification tasks given in Equation 3.


(3) $$\begin{array}{l} \mathrm{Accuracy}=\mathrm{Total}\;\mathrm{Number}\;\mathrm{of}\;\mathrm{Predictions}/\\ \;\mathrm{Number}\;\mathrm{of}\;\mathrm{Correct}\;\mathrm{Predictions} \end{array}$$

**Figure 9:**
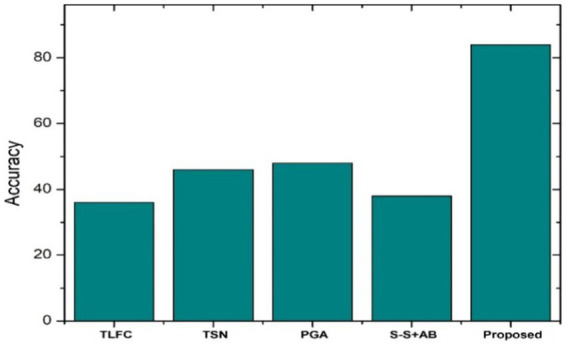
Accuracy score comparison.

Figure 9 depicts the accuracy scores for various summarization algorithms, including the proposed OAELSTM model on CNN/Daily Mail. The proposed model registers the highest accuracy score, substantially surpassing other algorithms such as the Token-level fact correction, Two-stage network, and Pointer generator. The accuracy score is critical as it measures the proportion of correct predictions made by the summarization model, which in this case, reflects the model’s ability to generate summaries that are well-aligned with the expected outputs.

The Token-level fact correction model shows the lowest accuracy, suggesting limitations in its summarization capabilities. The Two-stage network and Pointer generator models exhibit moderate accuracy scores, indicating room for improvement in summary generation. The chart clearly demonstrates the effectiveness of the proposed OAELSTM model in producing accurate summaries, which is a testament to the model’s robustness and the efficacy of the optimization techniques employed.

### 4.4 Comparative analysis and discussion

Our study’s findings, as illustrated in the ROUGE score comparison table, highlight the enhanced performance of the OAELSTM model over a range of established summarization approaches. Table 1 encapsulates ROUGE-1, ROUGE-2, and ROUGE-L scores, providing a comprehensive view of each model’s capabilities in generating summaries that align with human references.

**Table 1:** Comparison of the existing model with proposed with OAELSTM for ROUGE scores using CNN/Daily Mail dataset.

Model	Rouge-1	Rouge-2	Rouge-L
TLFC (Shin et al., 2023)	29.86	11.28	27.78
Seq-Seq + AB (Nallapati et al., 2016)	31.48	11.17	28.08
TSN (Dilawari et al., 2023)	37.16	17.81	33.83
PGA (See et al., 2017)	36.44	15.66	33.42
OAELSTM	44.34	20.24	35.89

The OAELSTM model demonstrates a notable advantage with the highest ROUGE-1 score of 44.34, indicating its strength in capturing key unigrams from the source text. Furthermore, it leads in ROUGE-2 with a score of 20.24, suggesting superior capture of bigrams or phrase-level information. In terms of ROUGE-L, which assesses the longest common subsequence, the OAELSTM model achieves a score of 35.89, underscoring its ability to maintain sentence-level structure and coherence.

The findings of the study, as illustrated by the ROUGE score comparison in Table 2, highlight the enhanced performance of the OAELSTM model over a range of established summarization approaches. The table encapsulates ROUGE-1, ROUGE-2, and ROUGE-L scores, providing a comprehensive view of each model’s capabilities in generating summaries that align with human references.

**Table 2:** Comparison of the existing model with proposed with OAELSTM for ROUGE scores with Gigaword dataset.

Model	Rouge-1	Rouge-2	Rouge-L
Concept pointer + RL (Wang et al., 2019)	38.02	16.97	35.43
Seq2seq + E2T_cnn (Amplayo et al., 2018)	37.04	16.66	34.93
JointParsing (Song et al., 2020)	36.61	18.85	34.33
OAELSTM	40.78	20.26	35.67

In particular, the OAELSTM model stands out with the highest ROUGE-1 score of 44.34, showcasing its effectiveness in capturing key unigrams from the source text. Additionally, it leads in ROUGE-2 with a score of 20.24, indicating superior capture of bigrams or phrase-level information. When it comes to ROUGE-L, which evaluates the longest common subsequence, the OAELSTM model achieves a score of 35.89, highlighting its ability to preserve sentence-level structure and coherence in the generated summaries.

### 4.5 Qualitative analysis

Table 3 showcases examples of summaries generated by the OAELSTM framework for articles from the CNN/Daily Mail and Gigaword datasets. The summaries provide a concise encapsulation of the primary content and context of the original articles. These summaries illustrate the effectiveness of the OAELSTM framework in distilling the essence of complex news articles into succinct, coherent summaries. The model’s ability to maintain the key points and factual accuracy of the original texts while condensing the information highlights its potential utility in diverse applications such as news aggregation, content curation, and information retrieval.

**Table 3:** Sample summary generated from OAELSTM framework.

CNN/Daily Mail—‘article’	Aluko nets winner with 10 min remaining at KC Stadium. Tomas Marek put visitors into shock lead after 2 min. Ahmed Elmohamady equalized for the hosts. Steve Bruce’s side now await Europa League play-off.
Gigaword—document	Australia’s current account deficit narrows sharply in the June quarter due to soaring commodity prices. Figures released Monday showed that the deficit was $1.2 billion US.

## 5 Conclusion and future work

The proposed study established the Optimized Auto Encoded Convolutional Neural Network (OAELSTM) with the Whale Optimization Algorithm (WOA) as a potent framework for text summarization. The OAELSTM model demonstrated a notable enhancement in summarizing texts, outperforming standard models in terms of Rouge score, accuracy, and specificity. The key innovation lies in its ability to effectively condense complex textual content into succinct, coherent summaries without losing the essence of the original texts. This breakthrough addresses critical challenges in natural language processing and opens new avenues in automated text summarization.

For future research, the focus will be on refining and expanding the capabilities of the OAELSTM framework. One area of interest is the incorporation of more advanced neural network structures to deepen the summarization context and accuracy. Exploring optimization techniques beyond WOA could further enhance feature selection and model tuning. Additionally, adapting the OAELSTM model for specific domains such as legal or medical texts can make it a more versatile tool. Assessing the model’s scalability for larger datasets and its practical application in various industries will also be crucial. These enhancements will not only bolster the OAELSTM framework’s effectiveness but also broaden its applicability in diverse real-world scenarios.

## Data Availability

The original contributions presented in the study are included in the article/supplementary material, further inquiries can be directed to the corresponding author.

## Footnotes

^1^https://huggingface.co/datasets/CNN_dailymail (Accessed January 2, 2024).

^2^https://huggingface.co/datasets/gigaword (Accessed January 2, 2024).
